# The Face of Workplace Violence: Experiences of Healthcare Professionals in Surgical Hospital Wards

**DOI:** 10.1155/2020/1854387

**Published:** 2020-05-28

**Authors:** Jenny Jakobsson, Malin Axelsson, Karin Örmon

**Affiliations:** ^1^Department of Care Science, Faculty of Health and Society, Malmö University, Malmö, Sweden; ^2^The Västra Götaland Region Competence Center on Intimate Partner Violence, Gothenburg, Sweden

## Abstract

**Background:**

Though workplace violence (WPV) is a global problem for healthcare professionals, research within in-hospital care has mainly focused on WPV in emergency healthcare settings. Thus, the number of qualitative studies that explores experiences of WPV in general hospital wards with a longer length of stay is limited.

**Aim:**

The aim of this study was to explore how healthcare professionals in surgical hospital wards experience and manage WPV perpetrated by patients or visitors.

**Method:**

The study applied a qualitative, inductive approach using focus group interviews for data collection. A purposeful sample of 16 healthcare professionals working in surgical wards was included. Data were analysed using a thematic analysis. *Findings*. The analysis resulted in four main themes: workplace violence characteristics, partly predictable yet not prevented, approaching workplace violence, and consequences from workplace violence. During the focus group interviews, the healthcare professionals described various acts of physical violence, verbal abuse, and gender discrimination perpetrated by patients or their visitors. Despite the predictability of some of the incidents, preventive strategies were absent or inadequate, with the healthcare professionals not knowing how to react in these threatful or violent situations. They experienced that WPV could result in negative consequences for the care of both the threatful or violent person and the other patients in the ward. WPV caused the healthcare professionals to feel exposed, scared, and unprotected. *Conclusion and clinical implications*. Exposure to WPV is a problem for healthcare professionals in surgical wards and has consequences for the patients. Preventive strategies, guidelines, and action plans are urgently needed to minimise the risk of WPV and to ensure a safe work and care environment.

## 1. Background

Workplace violence (WPV) perpetrated by patients or visitors in healthcare settings is a well-known and global problem with several negative consequences for healthcare professionals. Reports show that WPV increases the risk for ill-health, high staff turnover, and a deterioration in the quality of care [1]. Research has also shown that healthcare professionals refrain from reporting incidents because they feel that threats and violence have become a part of the job [2–4] or because they believe that reporting would not lead to any change [5]. This may indicate a high number of unreported incidents, thereby potentially leading to WPV towards healthcare professionals being underestimated or disregarded. Therefore, it is important to highlight this problem and its consequences.

Workplace violence is generally defined as “Incidents where staff are abused, threatened, or assaulted in circumstances related to their work, including commuting to and from work, involving an explicit or implicit challenge to their safety, well-being, or health” [1; p.3]. The particular characteristics of WPV towards healthcare professionals, perpetrated by patients or visitors, have been described in several studies [6–9]. Therein, the physical violence described includes patients punching, kicking, pushing, pinching, scratching, or spitting at the healthcare professionals. It also involves patients throwing things at them. However, verbal abuse has shown to be the most common act; examples would be patients or relatives yelling or swearing at the healthcare professionals, threatening them with physical harm, or pronouncing verbal threats or sexual harassment. According to a study by Avander et al. [10], verbal threats can also be expressed indirectly, for example, when disgruntled patients talk to relatives and friends on the phone in “a certain tone of voice” indicating that they hold the nurses responsible for matters about which they are dissatisfied.

In earlier studies [10–14], healthcare professionals in EDs and in trauma units who have been subjected to WPV describe that the experience gave rise to stress, insecurity, anxiety, and fear, all of which negatively affected their well-being. The stress caused physical symptoms such as headaches, stomach problems, sleeping disorders, loss of appetite, and difficulties in concentrating; this could also have a negative effect on the healthcare professionals' level of skill and efficiency at work. Moreover, stress relating to WPV affects not only the healthcare professionals' work life but also family and social life: it is difficult to ignore the stress, worry, and fear even on a day off. The healthcare professionals may even be afraid to leave the house [10–14].

Internationally and in Sweden, healthcare professionals working in emergency departments (ED), psychiatric and paramedic settings have been regarded as particularly at risk for exposure to WPV [15]. However, all healthcare professionals are at risk and not only those who work in EDs, within psychiatry or paramedics. In fact, in a study by Odes et al. [16], it was reported that professionals working in an ED were less likely to be physically injured by WPV than professionals working in an inpatient unit (i.e., a medical-surgical hospital unit). Healthcare professionals working in general hospital wards provide treatment and care to patients with a wide range of medical and surgical conditions, and available research indicates a high exposure to WPV. Hahn et al. [17] performed a systematic literature review on patient and visitor violence in general hospitals. In the review, it was reported that, on an average, 50% of the healthcare professionals had experienced verbal abuse, and 25% had been subjected to physical violence. The highest incidence was reported in surgical and medical wards and in intensive care units [17]. This finding is confirmed in a later meta-analysis by Spector et al. [18] who showed that an average of 26.7% of the nurses working in hospitals had been exposed to physical violence and 65.5% to nonphysical violence, ranging from rude remarks to serious verbal abuse. Despite the fact that WPV commonly occurs in general hospital wards, the majority of research has been conducted in settings regarded as particularly at risk, for example, in EDs [11–14]. Consequently, there is little understanding about how WPV affects the healthcare professionals in a setting where both care and work environment differ, as in surgical wards where patients can be cognitively affected by opioid medications or surgery, thereby acting in an aggressive manner. One of the largest differences is that patients admitted to surgical wards are cared for during a longer time than in, for example, EDs. Hence, the healthcare professionals on the ward must interact with a potentially harmful patient or visitor on a daily basis, and the risk of an incident will be constantly present. This may lead to an unhealthy work environment, but research addressing the problem in a surgical in-hospital setting seems to be sparse, and available research focuses more on the prevalence of WPV. This implies that there is a need to collect experiences derived from healthcare professionals working in surgical hospital wards, which can contribute to a deeper understanding of their situation. Such knowledge can be used in the development of hospital routines and interventions to prevent and handle WPV. Therefore, the aim of this study was to explore how healthcare professionals in surgical hospital wards experience and manage WPV perpetrated by patients or visitors.

## 2. Material and Method

A qualitative design with an inductive approach was used; data were collected by focus group interviews, as described by Krueger and Casey [19]. Given the dynamics and interactions of the focus groups, rich and varied descriptions of WPV were expected. Data were then analysed using a thematic analysis [20].

### 2.1. Setting

The study was conducted at a surgery department located in a university hospital in Sweden that serves about 1.7 million inhabitants. Approximately 350 assistant nurses, registered nurses, and physicians work at the department, which has a total of seven wards. The healthcare professionals provide both emergency and elective care to patients with a disease or injury in the upper or lower abdominal area. Patients are admitted to the wards if they have a disease or injury that requires either medical or surgical treatment, and they commonly suffer from pain or undergo surgery. Therefore, they can be cognitively affected due to anaesthesia, surgery, or opioid medication that can result in very aggressive behaviour. Healthcare professionals working in the surgical wards sometimes also care for patients who have been subjected to lethal violence, for example, gun violence. Often, those patients are still under threat at the ward and therefore require the presence of security guards or the police.

### 2.2. Participants and Recruitment

A purposeful sampling procedure that aimed for variation in age, gender, and profession was used [21]. Therefore, assistant nurses, registered nurses, and physicians working on the surgical wards were approached for recruitment.

The healthcare professionals were provided with verbal and written information by the researchers during their regularly scheduled workplace meetings, with a gatekeeper distributing information to those who could not attend. Moreover, a form was provided, in which the healthcare professionals could show interest in participating and give their contact information. Four focus group interviews were scheduled for the study, with the date, time, and place decided in advance, a strategy that is recommended by Krueger and Casey [19]. The first author contacted the professionals who showed interest in participating with an invitation to choose one of the sessions. The day before each session, a text message reminder was sent out. After recruitment in four of the seven wards, the four scheduled focus group interviews were saturated with participants. In total, 16 assistant nurses, registered nurses, and physicians from four of the seven wards participated. The background characteristics are presented in Table 1.

### 2.3. Data Collection Procedure

In April 2019, the focus group interviews were conducted in a secluded conference room near the surgical wards. The interviews were held by the researchers who took turns in being moderator who directed the discussions and assistant moderator who handled practical matters observed the interaction between participants and asked additional questions if needed. The four focus groups consisted of 2–5 participants and were of mixed professions given that the intention was to capture an interprofessional interaction. Before each interview, written consent was obtained along with the background characteristics of the participants. A semi-structured interview guide was used and included the opening question “Can you please explain what ‘threats' and ‘violence” means to you?” This was followed by four key questions addressing the participants' experiences of threats and violence. Additional probing questions were used to clarify or deepen the reasoning, for example, “Could you please explain…?,” “Could you tell more…?,” and “Is there anyone else who has had this experience?” The length of the interviews ranged from about 1–1.5 hours, and they were audio recorded and transcribed verbatim.

### 2.4. Data Analysis

Data were analysed using a thematic analysis, as described by Braun and Clarke [20]. This constitutes the analysis being conducted in five phases, with the sixth phase, described by Braun and Clarke [20], being the writing of the report.

The analysis commenced with a reading of the transcribed interviews individually to become familiar with the content (phase 1). During the next phase (phase 2), the data were reread, and a preliminary coding of the content was generated by tagging the text within the transcripts. Thereafter, the preliminary codes were compared and discussed in relation to the original data and the aim of the study. This generated a list of initial codes. In the following phase (phase 3), all the initial codes were written on self-adhesive notes and sorted into a map of preliminary themes and subthemes. The themes, subthemes, and codes were then reviewed to ensure that the themes accurately described patterns in the data (phase 4). In this phase, the transcribed data were reread. Also during this review, new codes were identified in the original text, and some codes were moved to another theme, while others were removed because they did not match the aim. In addition, some of the themes were merged together or separated into different themes. When the thematic map had been satisfactorily reviewed and refined, the title and essence of each theme was articulated (phase 5).

### 2.5. Ethical Considerations

The study was conducted in accordance with the Declaration of Helsinki [22] and approved by the Regional Ethical Review Board in Lund, Sweden (no 2018/800). In addition, the study was approved by the senior area manager of the surgery department who also facilitated access to the healthcare professionals.

The participants received both verbal and written information explaining that participation was voluntary, with the right to withdraw from the study at any time without explanation. The information was repeated verbally, and written consent was collected from the participants before each session. All data were handled confidentially.

## 3. Findings

Experiences describing the face of WPV at surgical wards resulted in four main themes and eleven subthemes (Table 2). In the following section, each theme is described in more detail.

### 3.1. Workplace Violence Characteristics

The workplace violence experienced by the healthcare professionals was characterised by physical violence, verbal abuse, and acts of gender discrimination. Physical violence was most likely to be perpetrated by patients with dementia, in emergence delirium after surgery, or in delirium related to drug or alcohol abuse. However, these types of incidents were experienced as unintentional and not personally directed; therefore, the healthcare professionals in general did not perceive them as particularly frightening:

With these patients who are confused or who have dementia, I think, yes, sure, you are shocked in that moment, and it remains for a short while. But afterwards, you let it go quite quickly because you feel that you understand the situation. And, yes, it was nothing personal ... there was no threat to me personally (Focus Group 2).

Nevertheless, situations could become unpleasant and sometimes dangerous, as healthcare professionals have been injured from attacks by delirious patients. Some of the most common violent acts committed against the healthcare professionals were when patients threw items such as shoes or medicine cups, hit them with their fist or cane, or pushed, bit, or grabbed them. More serious situations have arisen that involved confused or delirious patients who chased the healthcare professionals or broke windows, doors, or furnishings in acts of aggression or confusion.

Although acts of physical violence left the healthcare professionals feeling shaken, verbal abuse was perceived as potentially more frightening, as it could be more personally directed. Furthermore, the healthcare professionals described that the most serious and disrespectful form of verbal abuses involves threats made by relatives or other visitors, sometimes in combination with showing a knife, approaching the staff in a threatening way, or blocking the door. Such situations often appeared at the end of visiting hours when visitors were asked to leave. Healthcare professionals have been told by patients or visitors that they knew where they lived, when they quit their shift, and that family or friends would be looking for them after work. This causes feelings of not being safe, and healthcare professionals will sometimes call security for protection when walking to their cars after work.

Had the abuse only been about one's professional role, then I think it would have been easier to be less concerned. But when you start to feel that “Oh, they might actually know where I live,” then you feel a little vulnerable. I think that is more difficult and much scarier (Focus Group 4).

Not all verbal abuse was perceived as threatening. Frustrated patients or visitors and patients with substance or alcohol abuse craving for opioids among other things, might raise their voice and yell at the healthcare professionals, calling them incompetent, and using other disparaging words. However, such words were perceived as not personally directed, and they did not affect the professionals as much as verbal abuse of a more personal nature.

Some may often call you by your name ... which becomes much more personal. And it is also more uncomfortable compared to the usual (attitude that) “everyone here is a bitch” ... and well, you can somehow dismiss that in another way (Focus Group 4).

During the focus group interviews, the healthcare professionals also shared their experiences of gender discrimination behaviour from the patients and visitors. Acts of gender discriminatory were perceived as mostly, but not always, performed by male patients or visitors. This involved some female healthcare professionals being offended by sexually derogative words or their being touched in inappropriate places on the body.

Someone said that I should wear a tighter dress, something like that. It then becomes about how I am as a woman (instead of a professional), I felt ... Would he say these exact words to my male colleagues? Well, I do not know (Focus Group 3).

They also experienced that their professional skills and judgements were challenged because of their gender, thereby causing feelings of being disrespected and degraded.

### 3.2. Partly Predictable, yet Not Prevented

The healthcare professionals described that WPV sometimes could be predicted by early signs of impending aggression. Despite this, it was experienced that situations were not sufficiently prevented and that a lack of organisational guidelines left the healthcare professionals without knowing what course of action to take.

When caring for patients with dementia or patients with emergence delirium, the healthcare professionals explained that physical violence could be expected to a certain extent. Therefore, they were careful to monitor changes in the behaviour of these patients. Early signs could also be recognised in patients with substance withdrawal symptoms related to drug or alcohol abuse, as they may become irritated, raise their voice, or act angrily when they cannot obtain any more opioids, for instance. In those situations, the healthcare professionals emphasised the importance of preventing them from escalating, for example, by giving the patients the medical treatment to reduce their substance withdrawal symptoms. However, this request was not always recognised or the decision was made too late, thus putting the healthcare professionals in a vulnerable position because they eventually would have to handle the violent patient:

It was really problematic when we had many patients with alcoholism or other types of abuse, and the doctors did not respond when we pointed out they needed some (additional) treatment because they (the patients) were starting to get a little agitated, and so on. But it was just like—“No.” They ignored it (Focus Group 1).

Even though some threatening or violent situations were described as predictable, an atmosphere of unpredictability sometimes permeated the workplace, especially when caring for patients who act irrationally and unpredictably due to emergency delirium or substance withdrawal symptoms. Furthermore, when patients who had been subjected to gang-related violence were cared for, the presence of security guards and the police created an atmosphere in the ward that was described as “strange” and “uncomfortable.” Because of the nature of the patient's injuries (e.g., gang-related violence), the healthcare professionals were more wary when encountering the patient and the patient's visitors because of the potential threat they posed. One participant explains:

You do not know them, and you do not know their relatives. You do not know what kind of person they are and what they are capable of—you just do not know (Focus Group 2).

Although WPV was experienced by healthcare professionals as predictable to some extent, preventive strategies at the organisational level were perceived as absent or inadequate. They also felt that there was an unspoken attitude that one should accept threats and violence as being a part of the work. When WPV occurred more regularly, it became normalised, and the healthcare professionals described that they ceased seeing it as an event outside the norm of what is acceptable:

On becoming a nurse or assistant nurse, you note there is a culture that makes you feel that you should accept these things. Some things in healthcare you just have to deal with, like that … Some things are obviously not accepted. There are boundaries, but much is accepted by rationalising, (such as) “Yes, he is in pain” or “She is confused,” and so … I think there is a very clear culture where you just have to put up with certain things. And there is the feeling that you are a little troublesome if you make a big deal out of something you do not think is okay (Focus Group 3).

The healthcare professionals related that despite having access to relevant voluntary online education, they did not know what to do in a threatening or violent situation. One option was to call for a security guard. However, this was also perceived as false security because it takes time for the guards to come to the ward, and by then, the damage may have already been done. The healthcare professionals called for not only preventive strategies and clear guidelines but also for a joint effort within the organisation and in society in general to not accept WPV in healthcare.

### 3.3. Approaching Workplace Violence

Approaching WPV involves more than solely understanding the healthcare professionals' strategies to prevent incidents. It also concerns how they acted in situations that had become threatening or violent. The healthcare professionals explained that they applied individual strategies based on their own personal approach and work experience, while simultaneously working together and taking care of each other in the spirit of supporting colleagues with joint, contextual strategies. However, how they approached threatening or violent situations depended on the situation. This was often described as acting in an ad hoc manner. A primary strategy was to avoid conflicts. For potentially violent patients, the healthcare professionals tried to stay one step ahead by being observant and calm as they interacted with the patient or visitor. They also allowed the patients to decide more and were quicker to, for example, administer opioids if this was requested.

You try to encounter the patient calmly and maybe with a little understanding. You notice that the patient may start to feel pain … and instead of letting it get to the point where they have more pain, maybe you decide to administer pain relief earlier ... so it does not escalate. You try to always keep it at a manageable level (Focus Group 1).

When a threatening or violent situation occurred, the healthcare professionals felt unprepared. However, they tried to remain calm and solve the situation by reasoning with the patient or visitor. If that did not work, it was sometimes necessary to back off and let the patient or visitor get what they wanted, for example, by giving the patients more opioids or letting the visitors stay longer.

In threatening or violent situations, colleagues supported each other. For example, some explained how they would enter the patients' rooms in pairs or take turns seeing the patients:

If you have a patient and you feel threatened, afraid, or doubtful, then you bring it up in the group. Then you know that, okay, yes … you should not go in alone if the alarm goes; then we enter in pairs (Focus Group 2).

They also avoided unnecessary contact with the patient and the visitors. Moreover, calling a security guard for help only happened when the situation was out of control, for example, when patients with delirium or severe substance withdrawal symptoms raged and went on a physical attack. Sometimes, as the healthcare professional waited for the guard to arrive, they fled and locked themselves in the ward office.

### 3.4. Consequences from Workplace Violence

WPV was described by the healthcare professionals as having several negative consequences not only for them but also for both perpetrators and the other patients on the ward.

For the patients and the visitors who were threatening or violent, the care was described as affected both positively and negatively. On one hand, the patients received more attention, got help more quickly and were able to make more decisions themselves as a result of the healthcare professionals trying to prevent conflict or keeping a situation from escalating. On the other hand, the healthcare professionals avoided contact with the patients or their visitors, and consequently, there was a risk that the patients received less care. For example, they could miss out on information and important physical assessments.

You have to go in there and then … you really just try to think, “I have to do this … I will have to do it as quickly and smoothly as possible, and then I'll just try to leave the room again.” You might not hang around in there just to talk to the patient. Instead, you just go in, do it as quickly as possible and then leave (Focus Group 2).

The healthcare professionals maintained that the other patients in the wards became negatively affected by WPV. For example, because threatening or violent patients received more attention, other patients risked receiving less attention and information. They had to wait longer to receive analgesics, and physical assessments were delayed or even not performed. Another possible negative result is that the healthcare professionals are distracted and lack concentration, thereby increasing the risk of an error being made:

It is difficult to put the threatening or violent person aside and give the other patients the attention they need. You are still left in the other room. And it is possible to miss important things regarding the other patient (Focus Group 4).

Furthermore, the ward environment in general became affected, particularly with the presence of security guards and the police who make the patients feel worried:

The other patients do not know why there are a lot of police officers about. It may be that we have someone who is very dangerous in the room. They do not know, and it is clear that it is stressful for them (Focus Group 4).

It was explained that some patients had trouble sleeping and did not want to leave their ward rooms. There had even been occasions where a patient with emergency delirium after surgery had attacked another patient during night.

The healthcare professionals believed that WPV could not be completely avoided because they often had to be in proximity of the patients when caring for them. For example, they had to work physically close to patients with dementia despite the risk of being kicked or bitten. Therefore, these sort of threats and violence were considered as part of the job. Nevertheless, other situations of WPV had consequences for the healthcare professionals, as it gave rise to feelings of being exposed, vulnerable, unprotected, and hopeless:

I know I still have to take my shift tomorrow and take care of the patients in exactly the same way, but there is no plan, no guidelines or anything. And that is difficult to handle (Focus Group 4).

When a threatful or violent patient was cared for in the ward, especially longer periods, the healthcare professionals felt anxious, which could result in the reluctance to work. Some of the healthcare professionals had even recognised a pattern of avoidance among colleagues as they called in sick.

There is avoidance, as we talked about before. One might decide, “I'm sick today, I'm not going in. Then, I do not have to deal with this” (Focus Group 4).

Many healthcare professionals blamed themselves for being incapable of handling situations in a professional manner. They reflected on whether a man or an older, more experienced person would be better suited in their job:

I always wonder if a man or a larger person or an older person would have handled this situation better than I did. It becomes a question of not having the right personality to handle this profession, or so I can think. Or (is it) this place? I think, “Should I work elsewhere?” (Focus Group 4).

Physical violence, verbal threats, or dispraising words from patients or visitors were perceived as unacceptable by the healthcare professionals. Still, they often felt alone and vulnerable in these situations because they did not know how to act or what to do. If security guards or the police were present on the wards, they mainly stayed outside of the room, leaving the healthcare professionals alone with the patients or visitors. Furthermore, some of the security guards were regarded as physically too small and not commanding authority, which left the healthcare professionals feeling unprotected. For patients who had been involved in gang-related violence, the healthcare professionals reflected on the risk of getting in the way if someone should try to access the ward to harm the patient further:

What is so sick is that the patient who is the victim or the injured person is guarded by the police, and there they sit with bulletproof vests and weapons. And we are running around in our pajamas (referring to work clothes) (Focus Group 3).

## 4. Discussion

This study aimed to explore how healthcare professionals in surgical wards experienced and managed WPV perpetrated by patients or their visitors. During the focus group interviews, the healthcare professionals described WPV as consisting of physical violence, verbal abuse, and exposure to gender discrimination perpetrated by patients or the patients' visitors. Sometimes, threatening or violent incidents could be foreseen by early signs of aggression, but preventive strategies were experienced as absent or inadequate. Moreover, the healthcare professionals did not know what to do if they were exposed. WPV resulted in negative consequences for the healthcare professionals and for the care of both the threatening or violent person and the other patients on the ward.

The participants of this current study experienced being on the receiving end of physical violence, such as hitting, pushing, biting, and having objects thrown at them or deliberately broken in front of them. In addition, the participants encountered verbal abuse, which was described as patients or visitors yelling or calling the healthcare professionals disparaging words or speaking in a way that would be considered pronounced sexual harassment. Similar examples of WPV have also been described in earlier studies but in other settings [6–9]. Furthermore, physical violence is described in this current study as perpetrated by patients with dementia, emergence delirium after surgery, or delirium related to drug or alcohol abuse, while verbal abuse and gender discriminatory acts were pronounced mostly by seemingly rational patients or visitors. This is in line with Bigham et al. [2] whose research on paramedics gives the same description of WPV. Additional studies describe the perpetrators of WPV. In a study by Hyland et al. [6], a total of 91.6% of the participants in an ED with an experience of physical violence reported that the aggressor was a patient. This finding agrees with the result in the meta-analysis by Spector et al. [18] showing that the majority of physical violent acts (64.3%) were performed by patients. In contrast, in a questionnaire study by Kitaneh et al. [23] involving physicians and nurses in public hospitals, both physical and nonphysical violence—that is, verbal threats, verbal abuse, and sexual harassment—were shown to be the most frequently perpetrated by visitors or relatives of the patients. Other studies show a gender difference in the exposure of WPV, although research results are incongruent. For example, Acquadro Maran et al. [24] report that female healthcare professionals were more often exposed to WPV perpetrated by patients' relatives than their male counterparts, while Li et al. [25] found no significant gender differences. Edward et al. [26] reported that male nurses were at higher risk to the exposure of physical violence while female nurses had a higher risk of verbal abuse. This difference was also indicated in the study by Magnavita and Heponiemi [27], although it was not a statistically significant difference. In this current study, female participants had experiences of sexual harassment and reflected on whether a male colleague would have to endure the same sexually derogative words as they had experienced.

The current study shows how the healthcare professionals are affected by WPV differently, depending on the situation and the perpetrator. Hence, the healthcare professionals differentiated between types of WPV that frightened them and WPV that did not. For example, physical violence from patients with dementia or delirium was experienced as unintentional and perceived as not particularly frightening. This view has also been reported in other studies [3, 7, 10, 12]. In contrast, in the current study, personally directed verbal threats were perceived as more frightening, and such threats could be from both patients and visitors. The most frightening verbal threats were those that indicated that the professionals might be sought out and harmed outside of work. This finding is confirmed by a study by Hyland et al. [6], in which participants ranked verbal abuse as the most difficult to deal with compared to physical violence.

In earlier studies [10–14], healthcare professionals in EDs and in trauma units describe the consequences of WPV as feelings of insecurity, anxiety, and fear, and these fall in line with what the healthcare professionals in this current study also describe. However, the effect on the professionals' well-being in this current study was not highlighted as clearly as it has been in other studies. Other studies show conflicting results regarding the impact of WPV on health and well-being; for example, in a study by Shi et al. [28], participants who had experienced and been exposed to WPV were more likely to suffer from depression and anxiety symptoms than those who had not. On the other hand, Reknes et al. [29] showed that aggressive behaviour from patients or relatives had less of an impact on reported physical- and mental health-related quality of life than exposure to behaviour involving workplace bullying. Rather than focusing on well-being, some participants in the current study acknowledged that a higher rate of sick leave could be seen when the ward cared for a threatful or violent patient—sick leave that was discussed as not related to ill-health but rather to avoidance. In a study by Lancman et al. [30], ED staff experienced fear that the threatful or violent patient would return to the ED. In contrast to an ED, the healthcare professionals in a surgical ward know that the threatful or violent patient or visitor will be present over a longer time. It could be argued that the healthcare professional's choice to call in sick may be a consequence of organisational shortcomings in preventing and handling WPV, as the healthcare professionals also disclosed that they did not know what to do when such a situation occurred. Moreover, this type of “sick leave” will not be reported in statistical records as an effect of WPV or as an effect of the professional's assessment that there is risk of potentially being exposed to WPV, thus adding to the undervaluation of the problem.

The healthcare professionals in this current study perceived organisational strategies to prevent or handle WPV to be absent; moreover, they experienced the unspoken view that one should accept WPV as a part of the job. This unspoken view was also reported in a study by Wolf et al. [31] where ED nurses experienced that the hospital administration discouraged them from pressing charges against perpetrators. Additional studies on ED nurses [3, 6] have reported that WPV was regarded as part of a normal working day. Moreover, the nurses in the study by Hyland et al. [6] considered that if nobody got hurt, it was not worth reporting. It may be that this unspoken view, along with an undervaluation of the problem, could be a reason why strategies to prevent or handle WPV often seem absent or inadequate. This could lead to a vicious circle, with feelings of discouragement among healthcare professionals and a risk of an increased staff turnover. WPV has been described as positively correlated with a higher rate of turnover intentions and job burnout in physicians working in hospitals [32]. Moreover, research has shown that medical-surgical nurses with employers that did not listen to them regarding preventive strategies were less likely to feel safe at work [33]. Accordingly, a study by Lamont and Brunero [34] found a significant increase in nurses' confidence when dealing with patient aggression after attending a WPV training workshop.

In summary, it is clear that WPV prevention is a necessity, and it is equally important that employers acknowledge the problem. It is also essential that employers provide sufficient support for abused employees, but, even more importantly, employers should act more proactively to avoid WPV. The implementation of preventive measures to protect healthcare professionals so they feel safe and can do their job properly is an urgent need, in addition to the need to equip the healthcare professionals with strategies on how to act in situations of WPV.

In the focus group discussions, the healthcare professionals explained that WPV had consequences for the patients, both for the threatening or violent patient and the other patients in the ward. The former received more attention, and they got it faster because the healthcare professionals tried to avoid an escalating conflict. This was also described in a study by Avander et al. [10] involving trauma nurses who reprioritised their work to satisfy some patients' demands. This priority contrasted with the normal approach, whereby medical and nursing assessments determined how they worked. However, in this current study, the healthcare professionals described that, despite the increased attention they gave to the threatening or violent patients, they actually provided less care because they only did what was the most necessary and nothing more. In a study by Han et al. [13], nurses in an ED related that their coping strategies were to lower the standards of appropriate patient care, which is a strategy also described in a review by Lancôt and Guay [35] that shows the impact on the amount of time nurses spent with patients and what tasks were performed. According to Lancôt and Guay [35], not only were the threatening or violent patients affected but also the other patients in general. This was also related by the healthcare professionals in the current study. Given the great amount of attention directed to the threatening or violent patients, the other patients had to wait for their turn, thereby running the risk of not receiving, for example, analgesics or important physical assessments in time. In addition, the healthcare professionals found it difficult to leave a threatening or violent situation behind when they went to the next patient. This was also described by nurses in the study by Hassankhani et al. [11] who explained that WPV made them lose their concentration, thus affecting levels of skill and efficiency in the work, which in turn may compromise patient safety.

For this study, data were collected through conducting focus group interviews. Focus group interviews are often used within nursing research and are beneficial when investigating certain experiences [19, 36]. The method enables a group with a common experience to be heard, which was a focal point in the research conducted. Though talking about WPV may be sensitive and difficult, the opportunity to discuss experiences in a group could also be helpful for the individual participants. The focus group interview method was also considered pertinent because the professionals work closely with each other as a team and thus share experiences, though from different points of view. The focus group interviews conducted for this study were mixed regarding professions; however, the participants were used to work in interprofessional teams, which could prevent eventual problems with power hierarchy during interviews. The researchers also sensed that the participants felt comfortable to freely discuss the subject within the focus groups. In the results, the healthcare professionals pointed out that when threatening and violent situations occurred, they supported each other as colleagues. They all shared experiences of WPV regardless of profession, and this can be regarded as a factor strengthening the transferability of the result.

Nevertheless, it was challenging to recruit participants to the study. Although each focus group was intended to include approximately six participants, the workload of the professionals prohibited them from leaving the ward despite their interest in participating. Other reasons may be that certain threatening or violent incidents were experienced as part of the job and, therefore, not identified as WPV. Although every individual story is unique, a sample of 16 is small and only one physician participated, which could be seen as a limitation in this study. Nevertheless, we received deep and rich narratives concerning the healthcare professionals' experiences. The participants illuminated similar experiences, which justified the researchers decision to not continue recruitment. In addition, the findings in our study correspond well with findings of earlier studies. Therefore, we are confident that the result of our study is trustworthy and transferable despite the small sample.

## 5. Conclusion and Clinical Implications

This study found that healthcare professionals working on surgical wards are at risk to be exposed to WPV, much like those who work in settings that are traditionally regarded as particularly at risk. Therefore, the problem should be equally acknowledged. Though both healthcare professionals and patients were found to be negatively affected by WPV, there seemed to be insufficient organisational strategies to prevent and handle WPV. Given that WPV concerns a variety of threatful or violent acts perpetrated by different persons and during different circumstances, this study suggests that healthcare organisations should act urgently to address the problem by working out strategies to prevent WPV and by tailoring guidelines about what to do when a situation occurs. Regular training activities that include the interprofessional team and incorporating interprofessional simulation exercises within healthcare educations could be beneficial.

## Figures and Tables

**Table 1:** Background characteristics of the participants (*n* = 16).

Age, mean (SD)	37.3 (12.9)

Gender, *n* (%)	
Male	3 (18.8)
Female	13 (81.2)

Education/occupation, *n* (%)	
Assistant nurse	4 (25.0)
Registered nurse	11 (68.8)
Physician	1 (6.2)

Number of years working at the ward, mean (SD); min-max	7.0 (8.4); 0.5–32.0

*n* = number; SD = standard deviation.

**Table 2:** Overview of main themes and subthemes.

Main theme	Subtheme
Workplace violence characteristics	(i) Physical violence
	(ii) Verbal abuse
	(iii) Gender discrimination

Partly predictable, yet not prevented	(i) Early signs
	(ii) Unpredictability
	(iii) Organisational flaws

Approaching workplace violence	(i) Preventive strategies
	(ii) Acting during incidents

Consequences of workplace violence	(i) For the care of the threatening or violent patient
	(ii) For the care of the other patients
	(iii) For the professionals

## Data Availability

The data have not been made available due to ethical reasons. Given that the data are drawn from a small sample, there is a possibility that the participants could be identified if the original data were made available.

## References

[1] International Labour office/International Council of Nurses/World Health Organization/Public Services International (2002). *Framework Guidelines for Addressing Workplace Violence in the Health Sector*.

[2] Bigham B. L., Jensen J. L., Tavares W. (2014). Paramedic self-reported exposure to violence in the emergency medical services (EMS) workplace: a mixed-methods cross-sectional survey. *Prehospital Emergency Care*.

[3] Hogarth K. M., Beattie J., Morphet J. (2016). Nurses’ attitudes towards the reporting of violence in the emergency department. *Australasian Emergency Nursing Journal*.

[4] Knowles E., Mason S. M., Moriarty F. (2013). ‘I am going to learn how to run quick’: exploring violence directed towards staff in the emergency department: table 1. *Emergency Medicine Journal*.

[5] Arnetz J. E., Hamblin L., Ager J. (2015). Underreporting of workplace violence. *Workplace Health & Safety*.

[6] Hyland S., Watts J., Fry M. (2016). Rates of workplace aggression in the emergency department and nurses’ perceptions of this challenging behaviour: a multimethod study. *Australasian Emergency Nursing Journal*.

[7] Pich J., Hazelton M., Sundin D., Kable A. (2011). Patient-related violence at triage: a qualitative descriptive study. *International Emergency Nursing*.

[8] Renker P., Scribner S. A., Huff P. (2015). Staff perspectives of violence in the emergency department: appeals for consequences, collaboration, and consistency. *Work*.

[9] Speroni K. G., Fitch T., Dawson E., Dugan L., Atherton M. (2014). Incidence and cost of nurse workplace violence perpetrated by hospital patients or patient visitors. *Journal of Emergency Nursing*.

[10] Avander K., Heikki A., Bjerså K., Engström M. (2016). Trauma nursesʼ experience of workplace violence and threats. *Journal of Trauma Nursing*.

[11] Hassankhani H., Parizad N., Gacki-Smith J., Rahmani A., Mohammadi E. (2018). The consequences of violence against nurses working in the emergency department: a qualitative study. *International Emergency Nursing*.

[12] Ashton R. A., Morris L., Smith I. (2018). A qualitative meta-synthesis of emergency department staff experiences of violence and aggression. *International Emergency Nursing*.

[13] Han C.-Y., Lin C.-C., Barnard A., Hsiao Y.-C., Goopy S., Chen L.-C. (2017). Workplace violence against emergency nurses in Taiwan: a phenomenographic study. *Nursing Outlook*.

[14] Mento C., Silvestri M. C., Bruno A. (2020). Workplace violence against healthcare professionals: a systematic review. *Aggression and Violent Behavior*.

[15] Hallberg U. (2011). *Kunskapsöversikt- Hot Och Våld Inom Vård Och Omsorg*.

[16] Odes R., Hong O., Harrison R., Chapman S. (2020). Factors associated with physical injury or police involvement during incidents of workplace violence in hospitals: findings from the first year of California’s new standard. *American Journal of Industrial Medicine*.

[17] Hahn S., Zeller A., Needham I., Kok G., Dassen T., Halfens R. J. G. (2008). Patient and visitor violence in general hospitals: a systematic review of the literature. *Aggression and Violent Behavior*.

[18] Spector P. E., Zhou Z. E., Che X. X. (2014). Nurse exposure to physical and nonphysical violence, bullying, and sexual harassment: a quantitative review. *International Journal of Nursing Studies*.

[19] Krueger R. A., Casey M. A. (2015). *Focus Groups: A Practical Guide for Applied Research*.

[20] Braun V., Clarke V. (2006). Using thematic analysis in psychology. *Qualitative Research in Psychology*.

[21] Polit D. F., Beck C. T. (2008). *Nursing Research: Generating and Assessing Evidence for Nursing Practice*.

[22] World Medical Association (2013). World Medical Association Declaration of Helsinki: ethical principles for medical research involving human subjects. *The Journal of the American Medical Association*.

[23] Kitaneh M., Hamdan M. (2012). Workplace violence against physicians and nurses in Palestinian public hospitals: a cross-sectional study. *BMC Health Services Research*.

[24] Acquadro Maran D., Cortese C. G., Pavanelli P., Fornero G., Gianino M. M. (2019). Gender differences in reporting workplace violence: a qualitative analysis of administrative records of violent episodes experienced by healthcare workers in a large public Italian hospital. *BMJ Open*.

[25] Li M., Liu J., Zheng J. (2020). The relationship of workplace violence and nurse outcomes: gender difference study on a propensity score matched sample. *Journal of Advanced Nursing*.

[26] Edward K. L., Stephenson J., Ousey K., Lui S., Warelow P., Giandinoto J. A. (2016). A systematic review and meta-analysis of factors that relate to aggression perpetrated against nurses by patients/relatives or staff. *Journal of Clinical Nursing*.

[27] Magnavita N., Heponiemi T. (2012). Violence towards health care workers in a public health care facility in Italy: a repeated cross-sectional study. *BMC Health Services Research*.

[28] Shi L., Li G., Hao J. (2020). Psychological depletion in physicians and nurses exposed to workplace violence: a cross-sectional study using propensity score analysis. *International Journal of Nursing Studies*.

[29] Reknes I., Notelaers G., Mageroy N. (2017). Aggression from patients or next of kin and exposure to bullying behaviors: a conglomerate experience?. *Nursing Research and Practice*.

[30] Lancman S., Mângia E. F., Muramoto M. T. (2013). Impact of conflict and violence on workers in a hospital emergency room. *Work*.

[31] Wolf L. A., Delao A. M., Perhats C. (2014). Nothing changes, nobody cares: understanding the experience of emergency nurses physically or verbally assaulted while providing care. *Journal of Emergency Nursing*.

[32] Duan X., Ni X., Shi L. (2019). The impact of workplace violence on job satisfaction, job burnout, and turnover intention: the mediating role of social support. *Health and Quality of Life Outcomes*.

[33] Havaei F., MacPhee M., Lee S. E. (2019). The effect of violence prevention strategies on perceptions of workplace safety: a study of medical-surgical and mental health nurses. *Journal of Advanced Nursing*.

[34] Lamont S., Brunero S. (2018). The effect of a workplace violence training program for generalist nurses in the acute hospital setting: a quasi-experimental study. *Nurse Education Today*.

[35] Lanctôt N., Guay S. (2014). The aftermath of workplace violence among healthcare workers: a systematic literature review of the consequences. *Aggression and Violent Behavior*.

[36] Doody O., Slevin E., Taggart L. (2013). Focus group interviews in nursing research: part 1. *British Journal of Nursing*.

